# Validation of population-based disease simulation models: a review of concepts and methods

**DOI:** 10.1186/1471-2458-10-710

**Published:** 2010-11-18

**Authors:** Jacek A Kopec, Philippe Finès, Douglas G Manuel, David L Buckeridge, William M Flanagan, Jillian Oderkirk, Michal Abrahamowicz, Samuel Harper, Behnam Sharif, Anya Okhmatovskaia, Eric C Sayre, M Mushfiqur Rahman, Michael C Wolfson

**Affiliations:** 1 School of Population and Public Health, University of British Columbia, Vancouver, BC, Canada; 2 Arthritis Research Centre of Canada, Vancouver, BC, Canada; 3 Health Analysis Division, Statistics Canada, Ottawa, ON, Canada; 4 Epidemiology Division, Ottawa Health Research Institute, University of Ottawa, Ottawa, ON, Canada; 5 Department of Epidemiology, Biostatistics and Occupational Health, McGill University, Montreal, QC, Canada; 6 Department of Epidemiology and Community Medicine, University of Ottawa, Ottawa, ON, Canada

## Abstract

**Background:**

Computer simulation models are used increasingly to support public health research and policy, but questions about their quality persist. The purpose of this article is to review the principles and methods for validation of population-based disease simulation models.

**Methods:**

We developed a comprehensive framework for validating population-based chronic disease simulation models and used this framework in a review of published model validation guidelines. Based on the review, we formulated a set of recommendations for gathering evidence of model credibility.

**Results:**

Evidence of model credibility derives from examining: 1) the process of model development, 2) the performance of a model, and 3) the quality of decisions based on the model. Many important issues in model validation are insufficiently addressed by current guidelines. These issues include a detailed evaluation of different data sources, graphical representation of models, computer programming, model calibration, between-model comparisons, sensitivity analysis, and predictive validity. The role of external data in model validation depends on the purpose of the model (e.g., decision analysis versus prediction). More research is needed on the methods of comparing the quality of decisions based on different models.

**Conclusion:**

As the role of simulation modeling in population health is increasing and models are becoming more complex, there is a need for further improvements in model validation methodology and common standards for evaluating model credibility.

## Background

Computer simulation models have been used in health research and policy since the 1960s [1,2]. In a review of simulation modeling in population health and health care delivery prior to 2000, Fone et al identified 182 papers covering a wide range of topics, including hospital scheduling, communicable diseases, screening, cost of illness, and economic evaluation [3]. The authors noted that the quality of published papers was variable and the value of modeling was difficult to assess. One of the features distinguishing high quality papers from lower grade papers was more complete reporting of model validation [3]. While concerns have been raised about the role of modeling in guiding health policies [4,5], the number of published disease simulation models has grown dramatically in the past decade. This expansion has been fuelled by the increasing power and decreasing cost of computing infrastructure combined with the growing availability of population health data [5-7].

The methodology of model validation has been discussed extensively in the literature [3-15]. Sargent [8] described the general approach to "verification and validation" of computer simulation models and specific techniques that can be used for these purposes. Citro and Hanushek [9] discussed issues specific to the validation of microsimulation models in social policy and reviewed a number of validation studies. Morrison [10] provided a thorough review of the validation of the DYNACAN model, used to simulate changes in the Canada pension plan, and discussed many principles of model validation in this context. Weinstein et al [4] and the ISPOR Task Force on Good Research Practices [11] published guidelines for the development and validation of decision-analysis models in health policy. Philips et al reviewed 15 previously published guidelines on the validation of health technology assessment models and developed a comprehensive list of questions to be addressed [12]. The Environmental Protection Agency in the U.S. issued Guidance for the Development, Evaluation and Application of Environmental Models [15].

While the need for model validation has long been recognized in the literature, different methods and techniques may be needed for different models depending on the type of the model and the intended application [4,15]. Feuer et al distinguished between decision-analysis (cost-effectiveness) models and population-based "surveillance" models [16]. A surveillance model differs from decision-analysis models in that "rather than representing a hypothetical cohort, it models the population, that is, a collection of birth cohorts, over a specified period of time" [16]. These authors also drew a distinction between biological and epidemiological models. Biological models attempt to model the underlying disease process at the level of organs and tissues whereas epidemiological models simplify the process by focusing on the observable characteristics of individuals or groups [16]. In microsimulation models, the unit of simulation is the individual.

The purpose of this article is to review the principles and methods of validation applicable to population-based disease simulation models. The majority of published model validation guidelines were developed in the context of macro-level, cohort-based models designed as aids to decision making [11,12]. Population-based models have a wider range of application, including explanation and prediction of trends in disease frequency and estimation of unknown parameters through model calibration. We propose a comprehensive framework for the validation of such models. This framework addresses several gaps in the published validation guidelines and allows us to formulate specific recommendations for conducting model validation studies.

The focus of this review is on epidemiological microsimulation models of non-communicable diseases, rather than biological models or models that involve interactions between individuals (e.g., infectious disease models). Most of the examples come from our experience with the Population Health Model (POHEM) from Statistics Canada [17,18]. POHEM is a generic longitudinal microsimulation model of health and disease. The model simulates representative populations and allows the comparison of competing health interventions. Studies using POHEM-based models for breast, colon and lung cancer in Canada have been published [18-21], and a model of osteoarthritis has been developed recently [22].

## Model validation framework

In computer modeling, validity has been defined as "the degree to which a model or simulation is an accurate representation of the real world from the perspective of the intended uses of the model or simulation" [23]. However, validity defined in this way is often difficult to prove. It has been pointed out that model validation must be conducted continuously and should never be considered entirely complete [4,8,15]. From a practical perspective, models gain *credibility *among potential users by virtue of being carefully developed and thoroughly tested [10]. In assessing model credibility, the key issue is the amount of evidence, both theoretical and empirical, in support of the model's intended use. Consequently, we consider model validation broadly as the process of gathering such evidence.

Published guidelines differ in how they define the scope of model validation and what terms they use for various components of the validation process. For example, Sargent distinguishes between data validity,  validation, model verification, and operational validity [8]. The ISPOR guidelines consider model structure, data, and validation as criteria for model quality, with validation divided into internal, between-model and external/predictive validation [11]. Weinstein et al [4] used the term "verification" to mean determining that the model's inputs and outputs are consistent with actual data and accepted theories. Model corroboration in their framework involves comparisons between different models, whereas validation is equivalent to predictive validity testing. They use the term evaluation to encompass all these concepts [4]. A variety of other terms have been used in the validation literature [9-15].

The terminology of model validation can be simplified by recognizing that the evidence supporting a given use of a model can be obtained by examining: 1) the process of model development; 2) the performance of the model; and 3) the quality of decisions based on the model. All aspects of model development must be examined. These aspects include the underlying theories and assumptions, the definitions of key concepts, the content, the structure, the parameters, and the implementation of the model in software. Similarly, model performance must be examined comprehensively, starting with subjective plausibility (face validity), and including internal consistency, parameter sensitivity, between-model comparisons (model sensitivity), and external comparisons with historical and prospective data. Finally, important insights regarding model credibility can be obtained by considering the consequences of decisions based on the model (actual model applications). These sources of evidence are listed below.

1. Evidence from examining model development process

a. Conceptual model

b. Parameters

c. Computer implementation

2. Evidence from examining model performance

a. Plausibility (face validity)

b. Internal consistency

c. Parameter sensitivity

d. Between-model comparisons

e. Comparisons with external data

3. Evidence from examining the consequences of model-based decisions

## Evidence from examining model development process

### Conceptual model

The development of a disease simulation model usually starts with a conceptual description of the relationships between the condition of interest, its causes and its consequences. Sargent discussed the relationship between model development and model validation [8]. He defined conceptual model validation as "determining that the theories and assumptions underlying the conceptual model are correct and the model representation of the problem entity and the model's structure, logic, and mathematical and causal relationships are reasonable for the intended purpose of the model." The evidence of conceptual model validity tends to be qualitative and relies on the opinion of experts in the relevant fields [8].

#### Underlying theories

The conceptual description of the model should be based on an accepted theory of the phenomena under study [11,12]. For example, POHEM-based disease models apply concepts from several disciplines, such as demography, epidemiology, statistics, medicine, and health economics [17,18]. In contrast, cancer models developed as part of the CISNET program in the U.S. use biological theories of tumor growth [24], whereas the Archimedes diabetes model incorporates physiological theories of blood sugar level regulation and other biological concepts [25,26]. A number of authors have emphasized the importance of ensuring the validity of the underlying theories [11,15]. The lack of an adequate theoretical basis is a serious limitation that may compromise the model's credibility.

#### Definitions of variables

The conceptual and operational definitions of the variables in the model should be justified [4,8,11]. The appropriateness of a disease definition within a model depends on the disease, the purpose of the model, and availability of data. For example, the POHEM-based models of lung and colorectal cancer employ the same disease definitions as those used by Canadian cancer registries, which generally include pathological confirmation [19-21]. Other population-based models apply definitions based on clinical diagnosis, hospital discharge information, self-report, or administrative data [22,24-28]. Evidence that the definition used is acceptable should be provided and may include a reference to published and/or generally accepted clinical criteria or results from validation studies. For example, the authors of the POHEM osteoarthritis (POHEM-OA) model performed a validation study to assess the sensitivity and specificity of an OA definition based on physician billing data against radiographic and clinical diagnosis [22]. Definitions of other variables, such as disease risk factors or quality or life outcomes, should also be provided and should be consistent with the definitions used in the literature. For instance, quality of life outcomes in the POHEM-OA model are based on a well-established instrument, the Health Utilities Index [22].

#### Model content and structure

Epidemiological simulation models usually represent causal relations between etiological factors and health conditions and between prognostic factors and health outcomes [16]. These relationships can be very complex (web of causation). The task for model developers is to determine those aspects of the causal web that are necessary and feasible to simulate. Model assumptions should be presented clearly, including a description of the expected strengths and limitations of the model for a range of potential applications. Determining which assumptions are most critical often depends on the purpose of the model (e.g., prediction, explanation, decision analysis) [4,15].

Evidence that the model is sufficiently complete and that the relationships between the variables are specified correctly should come from both theory and empirical data [8,11,15]. Incorrect model specifications may involve, for example, assuming causality between two variables that are merely correlated, ignoring a causal relationship, or assuming an incorrect direction of a causal effect. If some risk factors, intermediate variables, or interactions are omitted, explanation should be given as to why this omission is acceptable and does not invalidate the results. For example, the POHEM-OA model includes age, sex and BMI as risk factors for OA [22]. Although other factors have been reported in the literature, they have not been incorporated into the model because the evidence for their causal association with OA is inconclusive (e.g., education, physical activity) or because data on their distribution in the population are insufficient (e.g., family history, joint injury). Omitting the latter variables is unlikely to affect model performance since family history is not a modifiable factor and joint injuries are relatively rare.

The structure of a simulation model is often presented graphically. However, there is currently no standard format or generally accepted guideline for the graphical representation of epidemiological microsimulation models. Diagrams showing transitions between disease states typically used to describe macro-level models or data-flow diagrams common in computer science are not ideal to represent epidemiological microsimulation models in which the key consideration is the causal relationships between variables. For such models, diagrams based on graph theory [29], in which an arrow or arc linking two variables in the model indicates the assumed direction of a causal effect, may be more suitable.

Additional complexities arise in models in which different sets of relationships are defined for different subsets of the population. For example, in a model of breast cancer that simulates the risk factors for the disease as well as prognostic factors in different stages of the disease, the effects of prognostic factors apply only to those with breast cancer (or a specific stage of breast cancer). To represent such models, it may be helpful to use multiple diagrams or multilayer computer-based interactive diagrams [30].

### Parameter validation

Model developers are familiar with the popular dictum "garbage in, garbage out". Clearly, providing evidence of parameter validity is important for establishing model credibility [10]. However, population-based microsimulation models of chronic diseases may include thousands of parameters [10,16,18,22]. For example, the parameters in POHEM-based models include: age/sex-specific mortality rates and birth rates in the population; age/sex-specific prevalence of the risk factors; effects of demographic variables on the probability of exposure to each factor; disease incidence by age and sex; causal relative risks (hazard ratios) for several risk factors with multiple levels and interaction effects; impact of various prognostic factors on disease progression and case fatality; probability of receiving different treatments conditional on patients' characteristics; the effects of each treatment on disease progression, complications, quality of life, and case-fatality; as well as economic parameters, such as cost of treatments according to disease stage and demographic variables [19-22]. Because of the number of parameters involved, it is not possible in such models to provide detailed and succinct summary of the evidence of validity for each parameter. The amount and type of evidence that should be provided will depend on the type of parameter, its source and method of estimating the parameter value, available data, and the importance of the parameter in a given application of the model.

Evidence of parameter validity should include considerations of possible bias as well as uncertainty in estimating a given parameter. Such evidence generally comes from examining the process used to derive a value for the parameter (primary source, method of derivation), and comparisons with data from other sources. The main sources of parameter values are: 1) expert opinion, 2) published estimates, 3) analyses of existing data, 4) collection and analysis of new data, and 5) model calibration. The decision as to which source to use often depends on data availability. When more than one source is available, the type of parameter, its likely impact on model output, quality of the source, and costs of parameter derivation are key considerations [8-11].

#### Parameters obtained from experts

While expert opinion is a legitimate method of obtaining parameters [11], the decision to use experts to estimate a parameter should be justified and the process of obtaining the parameters should be described. In our view, such description should include the number of experts, their area or expertise, what questions were asked and how the responses were used to identify a specific parameter value. Ideally, model developers should apply established methods of soliciting opinions from experts, such as Delphi or nominal group techniques [11]. The plausibility of the parameter value(s) must be assessed and deemed acceptable by independent experts. If other sources for a given parameter are available, comparisons should be made and the differences explained.

#### Parameters obtained from the literature

Parameters may be obtained from published or unpublished sources (e.g., government reports). Usually, the best source is a meta-analysis published in a peer-review journal. If several estimates for a parameter have been published but a meta-analysis has not been carried out, model developers may decide to perform their own meta-analysis or combine information from several sources in other ways [11,23,31]. The methodology for such derivation must be justified and explained in a manner likely to satisfy a scientific peer-review.

The results of meta-analyses are typically conditional on the estimates reported in individual studies, some of which may be seriously biased. For example, a review of survival studies in various cancers found that only 5% of studies that relied on the popular Cox proportional hazards (PH) model tested the crucial PH assumption that the effects of prognostic factors remain constant over time [32]. Yet, a failure to account for violation of the PH assumption may lead to biased prognosis and incorrect conclusions [33]. Therefore, a single high-quality study may be a preferred choice in some situations. For example, in the Coronary Heart Disease Policy Model, the risk of CHD was based on data from the Framingham Heart Study [31]. A single high-quality study may also be used when multiple estimates for a given parameter are not available. For instance, a POHEM-based breast cancer model used data from the Breast Cancer Prevention Trial to assess the benefits of tamoxifen in the prevention of breast cancer [19].

If high-quality studies (e.g., large randomized trials or cohort studies published in respected journals) are not available, other data sources may be used. However, simply providing a reference to a published source is generally not sufficient. The quality of the study should be ascertained and the selection of the source justified. If an unpublished source is used, evidence of validity should include a description of the study and an assessment of its quality. A comparison with alternative sources or data should be made and discrepancies explained or determined to be acceptable. If alternative sources are not available, the plausibility of the parameter values should be assessed by experts in the field. Data obtained directly from the literature may not be sufficient if important details are omitted. For example, the dose-response relationship is not fully delineated, subgroup analyses are not reported, not all age groups are analyzed, or non-significant results are omitted [12,23]. Model developers may have to apply analytical techniques such as smoothing, interpolation and extrapolation to derive the parameters, for example, assuming a linear or exponential dose-response relationship [15].

#### Parameters obtained from data analysis

Descriptive parameters in population health models may be derived directly from an existing database. For example, some POHEM-based models use the Canadian Community Health Survey as the baseline population, vital statistics as the source of mortality parameters, and an administrative health database for incidence and health care utilization rates [19-22]. Validity of the database must be ascertained as the accuracy of the data varies across different variables and types of parameters. Evidence of validity may come from previous validation studies or from new studies conducted specifically to assess validity against other sources, including other databases, the literature, as well as new analyses of the data. In POHEM-OA, for instance, age/sex-specific estimates of OA incidence derived from administrative data were compared with published estimates of the incidence of radiographic and symptomatic OA of different joints and self-reported arthritis incidence from population surveys [22].

Identified parameter values may be used directly or may undergo some modification or adjustment prior to incorporation into a simulation model. Morrison describes examples of adjustments and corrections applied to the parameters derived from a database [10]. For example, a public use database may include age as a grouped variable while the model requires exact ages. The exact age would thus be imputed based on the distribution of age in each category observed in a different database [10].

If parameters are derived through an analysis of existing or newly collected data, evidence of validity should be equivalent to that required for a publication in a scientific peer-review journal. In some cases, the results of such analyses are in fact published [34]. However, microsimulation models may require numerous and extensive analyses with hundreds of output tables. Even though appendices with additional data are increasingly posted online, publication of all analyses and model parameters in peer-review journals is generally not feasible. Also, the type of analysis required for a simulation model may not be suitable for publication in a medical journal. For example, the POHEM-OA model required an estimate of the overall effect of BMI on OA of any joint. Yet, for a clinical audience, joint-specific estimates would have been more interesting and more likely to be published.

Even if the results are not intended to be published, model developers must conduct data analyses that can stand up to scrutiny equivalent to that required for publication in peer-review journals. This includes not only proper statistical methods of analysis but also considerations of selection and measurement bias in the data, as well as confounding and presence of intermediate variables when assessing causal associations. In addition, it is often necessary to consider dose-response relationships, time-dependent effects, and interactions. The results should be compared with estimates from other sources and expert opinion.

Finally, it is critical that the estimates be generalizable to the population being modeled. If parameters are estimated from studies in non-representative populations, evidence to support generalizability should be provided. For example, the effect of surgical treatment on health outcomes in the POHEM-OA model was obtained from a cohort study in a single treatment centre because the study included multiple measurements of health utilities (key outcome in the model) prior to and following surgery. Although this was not a general population sample, the approach could be justified by the fact that this type of surgery has been highly effective in multiple studies and across different samples [35].

#### Parameters obtained through model calibration

Calibration of the model involves the estimation of unknown model parameters, so that the aggregate output from the model is consistent with external (target) data [15,16,36]. For example, in POHEM-OA, age/sex-specific incidence rates of OA in the reference population (those with normal BMI) have been calibrated to administrative data [22]. Calibration sometimes involves simultaneous searches for multiple parameters using multiple targets. A review of calibration methods in cancer models recently published by Stout et al included a 16-question checklist for reporting calibration studies. The following elements should be reported: target data, search algorithm, goodness-of-fit metrics, acceptance criteria, and stopping rule [36]. It should be pointed out that model calibration methodology is evolving and there is no consensus at this time on how calibration should be carried out [36,37]. Nevertheless, a detailed description of the calibration procedures would enhance model credibility. Furthermore, plausibility of the parameters derived through calibration should be evaluated by experts and their values compared with external data, whenever such data are available.

### Computer implementation

#### Selection of model type

Published validation guidelines do not specify how the appropriateness of model type should be determined. Similar modeling objectives can often be achieved with different types of simulation models [15]. For example, a CISNET simulation study of breast cancer mortality was performed with seven different models [38]. Providing a justification for the selected model type improves model credibility. Specifically, a stochastic rather than a deterministic model may be appropriate if modeling the full distribution of an outcome is important [39]. If a microsimulation approach is used, the need for additional complexity compared with a simpler macro-level model should be explained. Similarly, within the microsimulation approach, there should be a justification for choosing between discrete and continuous time models, as well as between "agent-based" models in which the individuals interact with each other and models that do not allow for such between-subject interactions. For example, unlike most infectious disease models, POHEM does not model contacts between persons because such interactions are irrelevant for describing the frequency, treatment and outcomes of non-communicable conditions. Whether or not the type of model is appropriate for a given application should be determined by independent experts.

#### Simulation software

Simulation models can be developed using a general-purpose programming language such as C++ or Java, more specialized languages such as MATLAB [40] or R [41], or software toolkits specifically designed to facilitate the construction of simulation models by providing graphical interfaces to other programming languages. For example, the source code for POHEM is written in Modgen, a C++-based simulation modeling language developed at Statistics Canada [42]. Wikipedia lists 67 agent-based modeling toolkits [43] and 22 discrete event simulation toolkits [44]. Some of the most commonly used agent-based modeling software has been reviewed by Railsback et al [45].

However, information on the relative merits of different software for microsimulation modeling is limited. More specifically, current model validation guidelines do not address the issue of selecting the most appropriate simulation platform. Advantages of using specialized simulation software for model development include greater model transparency and less opportunity for mistakes, thus improving model credibility. However, disease simulation models may be written in a general-purpose language because existing toolkits are not flexible enough, the specialized software may not execute efficiently, or the programmers are not familiar with the available simulation software. Model developers should provide information on the programming language and software used, and the reason for their choice.

#### Computer program

Implementation of a model in software involves many programming decisions. Even within a given type of model and simulation software, some development approaches are more appropriate (e.g., more efficient, less prone to errors) than others [8,46]. As part of model validation, programming experts not involved directly in model development should evaluate the key decisions and approaches used.

Published guidelines underscore the need for a careful examination (verification) of the computer program [8,10-12]. Debugging of the program involves various tests that can be performed to identify coding errors and other problems with the implementation of the model. Static techniques require the programmer to examine the structure of the program whereas dynamic techniques involve running the program or its components and comparing the results with an expected pattern of results [8]. More advanced methods of model specification and verification, known as formal methods, are based on theoretical concepts in computer science [46]. While relatively complex and expensive to implement, such methods are useful for models in which the costs of a mistake are extremely high. Some of the questions that need to be asked when validating the computer program are listed by Morrison [10]. Debugging is usually performed by the model development team. Evaluation of the source code by external experts is rare because of intellectual property concerns [11,15]. This practice, however, adds to the impression of simulation models as "black boxes". Documenting the results of program debugging tests and making the mathematical equations underlying the model open to scrutiny by external experts would improve model transparency and should become a standard practice [11].

## Evidence from examining model performance

If we were certain that the conceptual model, its parameters and computer implementation were all free of errors, there would be no need to examine model output as part of model validation. Unfortunately, no model is perfect. By definition, all models involve assumptions and simplifications that lead to discrepancies between the model output and the real world. Examining the output is thus an integral part of model validation. Sargent refers to this aspect of validation as examining "operational validity" of the model and defines it as "determining that the model's output behaviour has the accuracy required for the model's intended purpose over the domain of the model's intended applicability" [8]. This aspect of validation is also referred to as internal and external consistency [12], model verification [7], and external and predictive validity testing [11].

### Plausibility

The first step in examining model performance is usually the assessment of output plausibility (face validity), which consists in asking subject-matter experts if the model output appears reasonable and makes intuitive sense [7,8]. This involves comparisons of model output with expectations based on general knowledge and understanding of the modeled phenomena. Plausibility should be evaluated for a wide range of input conditions and output variables over varying time horizons [8].

Although the criteria for model plausibility are subjective and arbitrary, this is an important step in evaluating model performance and may point to potentially serious problems with the model [11,12]. Some results may be clearly implausible even to non-experts; however, plausibility is best assessed by persons with expert knowledge in the area of model application. For example, estimates of disease prevalence may appear unreasonably high or low, a curve describing a secular trend may have an improbable shape, or the impact of changing some input parameters may be opposite to what would be expected. In a preliminary version of POHEM-OA, the authors observed an implausibly rapid increase in OA prevalence in the first 20 years of the projected trend. Further analyses revealed that this problem was caused by a discrepancy between baseline prevalence and incidence of the disease estimated from administrative data. Subsequently, a better estimate of baseline prevalence was derived using simulation modeling [22].

### Internal consistency

Internal consistency is assessed by considering functional and logical relationships between different output variables [5,16]. The relationships between trends in disease incidence, prevalence, mortality and other health outcomes generated by the model should be consistent with theory. For example, assuming no change in case-fatality over time, one may be able to estimate the expected change in mortality associated with a given change in disease incidence. In POHEM-OA, which assumes no effect of OA on the risk of death, the authors assessed the relationship between the incidence, prevalence and duration of disease [22]. A lack of internal consistency usually suggests errors in the formal logic of the model or its implementation in software [12].

### Parameter sensitivity analysis

The definition of sensitivity analysis varies between authors. In most published guidelines, sensitivity analysis is regarded as a method of assessing the impact of parameter uncertainty on model output [10,13,14]. Citro and Hanushek define sensitivity analysis more broadly as "a technique that measures the effect on model output of alternative choices about model structure" [11]. Furthermore, some authors differentiate between analyses aimed at estimating a confidence interval around the output (uncertainty analysis) and those aimed at apportioning uncertainty in the output to different sources (sensitivity analysis) [15,47]. In this article, we use the term parameter sensitivity when discussing sensitivity analysis as a method of quantifying the impact of parameter uncertainty. Assessing the impact of uncertainty about the conceptual model (structural uncertainty) or computer implementation is discussed under "between-model comparisons".

Several methods of sensitivity analysis have been described [15,47]. The impact of parameter uncertainty is usually evaluated by running the model repeatedly while varying the values of the parameters. Parameter values can be varied systematically, either one at a time or in combination (deterministic methods), or sampled randomly from a univariate or, in the case of correlated parameters, joint multivariate probability distribution (probabilistic methods) [15,47]. In stochastic models, it is also important to assess the amount of stochastic variability (Monte Carlo error) through multiple runs of the model. A high amount of variability may cause the model's results to be questionable or seriously limit their practical utility. Stochastic error can be reduced by increasing the size of the simulated population [48], an option that becomes increasingly viable with continuing progress in computing resources.

Sensitivity analysis is an important component of model validation. According to the ISPOR guidelines, all modeling studies should include extensive sensitivity analyses with respect to key parameters [11]. The ISPOR guidelines consider either deterministic or probabilistic sensitivity analysis as appropriate. Philips et al recommend probabilistic analysis as the preferable method of handling parameter uncertainty [12]. Cronin et al provided a detailed discussion of probabilistic sensitivity analysis in the context of disease microsimulation models [49].

The large number of parameters in many microsimulation models makes sensitivity analysis a challenging task. As previously mentioned, some POHEM-based models may include thousands of parameters. For such models, expert opinion and preliminary screening tools, including graphical methods, can be used to select the most influential parameters [15,50]. More intensive sensitivity analysis methods are then applied to the smaller set of parameters. Among the screening tools, the most common approach is one-at-a-time analysis [51]. Useful graphical methods include tornado graphs, radar graphs, matrix and scatter plots and cobweb plots [52]. When certain parameters in the model are estimated through calibration, modifying the values of other parameters may require a recalibration of the entire model. In this case, the analysis will permit assessing the sensitivity of model output to various combinations of parameters that produce results consistent with the calibration data.

### Between-model comparisons

There is agreement in the literature that comparing the results of different models provides important evidence of validity and increases model credibility [7-13]. The ISPOR guidelines refer to this activity as model corroboration (or convergent validity) and state that "modellers should cooperate in comparing results and articulating the reasons for discrepancies" [11]. They also emphasize that alternative models should be developed independently of each other.

Alternative model structures and assumptions are increasingly considered a source of variation in model output that needs to be evaluated and quantified in a systematic way [9,47,53]. For example, Bojke and colleagues identified four major types of uncertainties in cost-effectiveness models [53]. However, systematic approaches to identifying and analyzing structural uncertainty in more complex population-based models of chronic diseases are lacking. Nevertheless, between-model comparisons can provide important insights into the impact of different approaches to model building on simulation results. Examples of successful between-model comparisons include the use of seven independently developed CISNET models to assess the impact of breast cancer screening and chemotherapy on breast cancer mortality [38] and the comparison of different diabetes models known as the Mount Hood Challenge [54].

To assess the sensitivity of model output to alternative model structures, it may be useful to modify different aspects of the model one at a time. For example, a new risk factor may be added to an existing model, different assumptions about causal effects can be incorporated, or a different type of computer model can be built using the same conceptual structure and parameters [9]. Simulations can be run for a range of plausible assumptions about the distribution of the omitted variable and its relationships to other variables. It is important to note that models should only be compared when they generate comparable outputs. For example, a population-based model and a cohort-based model of OA would produce different measures of disease burden and such models should not be compared.

### Comparisons with external data

There is some disagreement in the literature regarding the use of external data in model validation. Sargent advocates a formal external validation on a subset of the data that has not been used in model development, whereby statistical and graphical techniques are employed to compare actual observations with predictions from the model [8]. Similarly, Feuer et al state "after the model is calibrated, other data sets must be used to validate the model, that is, to evaluate whether the model produces results that match observed data not used for the calibration process" [16].

In contrast, Weinstein et al consider all comparisons of model output with existing external data as part of model calibration and refer to this process as model verification [4]. They reserve the term validation for comparisons with future events, observed after the model has been developed and calibrated. Philips et al emphasize that all available data should be used in model development and data should not be withheld for the purpose of external validation [12]. In other words, the model should be consistent with all relevant data available at the time it is developed. These authors limit the notion of external consistency to making sure that the results make intuitive sense and seemingly counterintuitive results are explained [12].

#### Predictive validity

Weinstein et al define (predictive) validation as comparisons with prospective data (future events) [4]. After reviewing the conditions required for predictive validation, such as constancy of the situation over time and across variations of conditions not specified in the model as well as availability of sufficient data to make predictive tests, they concluded that few models in healthcare could ever be validated for predictive use. This, however, does not disqualify such models from being used as aids to decision making [4]. Philips et al state that since a decision-analytic model is an aid to decision making at a particular point in time, there is no empirical test of predictive validity [12]. From a similar premise, Sculpher et al argue that prediction is not an appropriate test of validity for such models [13].

This issue is discussed from a slightly different perspective by Citro and Hanushek [9], who underscore the distinction between (a) predicting differences in outcomes between different scenarios or policies versus (b) predicting absolute levels of the outcome in the future. This distinction is important because errors that affect different scenarios equally may cancel each other. In decision-analysis models, absolute levels of the outcomes are usually less important than comparisons between alternative policy options. However, these authors also point out that comparisons between scenarios may be affected by errors in absolute predictions [9].

In contrast with decision-analysis models, population-based simulation models are often used to explain past trends in disease frequency or mortality and/or predict future trends [3,15,16,22,24,28,31]. As emphasized by Weinstein et al, predictions from such models should be treated with caution and regarded as conditional on model assumptions [4]. Prospective validation is rarely feasible because the time horizon for such models is often too long. In lieu of prospective validation, ex-post forecasting and backcasting based on historical data should be used to support predictive validity [9]. Interpretation of the differences between the observed and predicted values may be facilitated by the knowledge of uncertainty bounds of the model output and the distribution of the variable(s) of interest in the population. What constitutes a "close" prediction will depend on the practical implications of prediction errors in a specific application of the model and should be established in consultation with the user of the model.

When historical data are used for external validation, they should be different from the data used to populate and calibrate the model [4,9,16]. Thus, withholding part of the data for predictive model validation is appropriate. For example, in the context of cancer models, incidence data could be used for calibration and mortality outcomes for validation [16]. Alternative approaches that could approximate the expected results of external validation and reduce the need for withholding data are cross-validation and bootstrap re-sampling [55,56]. In these approaches, all available data can be used for model calibration. Validation is accomplished by running the model on multiple subsamples from the target datasets. In cross-validation, the data are split into multiple samples, whereas bootstrapping is based on re-sampling with replacement. Although well established as methods of validating statistical prediction models [55,56], cross-validation and bootstrapping have not, to our knowledge, been applied to validate disease simulation models.

In the validation of population-based models, reliable data on the effects of health policies may be more difficult to find than data on natural trends in disease frequency or mortality. Randomized community trials of population-based interventions are relatively rare, whereas data on trends in disease incidence or mortality may be available from cause-of-death statistics, national registries (e.g., cancer registries) and large administrative databases. On the other hand, data on treatment effects, relevant to many decision-analytical models, may often be obtained from randomized trials. Both types of data have been used for external model validation. For example, the CISNET cancer models have been validated by comparing ex-post predicted and observed historical trends in cancer incidence and mortality [16,24,38]. The developers of the Archimedes diabetes model compared model predictions against observed results from clinical trials [25,26].

## Evidence from the consequences of model-based decisions

Sculpher et al [13] considered the question whether cost-effectiveness models can be regarded as *scientific *models. They argued that randomized trials (even ideal pragmatic trials) and observational studies do not provide a valid test of model predictions. The reason is that cost-effectiveness models are developed to improve decision-making, not to predict future events. However, they concluded that such models are scientific because they could be falsified, at least in principle, by comparing the consequences of decisions that are based on models and decisions that are not [13].

The above argument essentially equates model validity with usefulness [13]. Yet, how usefulness of a model should be defined and measured is not clear. Ideally, criteria for the acceptability of a simulation model to the intended user should be specified in advance [15]. While usefulness is related to the accuracy of projections generated by the model [9], the level of accuracy needed for the model to be useful will depend on the specific application. For decision-analytical models, uptake of a given model by policy makers could be considered an indirect indicator of usefulness. Weinstein et al reviewed the applications of simulation models by the military, their role in influencing environmental and public health policies, and their use in the formulation of clinical practice guidelines [4]. The authors point out that the widespread use of models in these areas demonstrates that models "are perceived as valuable by organizations entrusted with our healthcare dollars"[4]. However, it does not necessarily prove they lead to better decisions. In principle, the impact of models on the quality of decisions could be evaluated directly in a variety of ways, including subjective and objective measures. More research on how these types of evaluations should be conducted is needed.

## Conclusions

In this paper we reviewed the types of evidence that can be used to support the use of population-based disease simulation models. Although a number of checklists for model validation have been published, important gaps remain in the validation literature. Our framework for model validation includes the assessment of all aspects of model development and implementation, examination of model performance, and evaluation of decisions based on the model. Recommendations for model validation based on the proposed framework are presented in Table 1. These recommendations are intended to be used primarily as general guidelines rather than a quantitative assessment tool. An optional scoring rule that can be used to assess the degree of validation is described in the Appendix. However, the scoring rule has not yet been validated and should be regarded as preliminary. We should emphasize that not all types of evidence listed in Table 1 apply to all models and that some validity criteria may be more important than others, depending on the specific application. For example, recommendations regarding parameters derived from experts would not apply to models in which such parameters are not used.

**Table 1:** Recommendations for gathering evidence of model credibility

Evidence from examining model development process	
Conceptual model	

Underlying theories	The conceptual model should be based on an accepted theory of the phenomena under study. The lack of an adequate theoretical basis is a serious limitation that may compromise the model's credibility.

Definitions of variables	Definitions of the variables in the model should be justified. Evidence that the definitions are acceptable should be provided (e.g., a reference to published and/or generally accepted clinical criteria or results from validation studies).

Model content and structure	Evidence should be provided that the model is sufficiently complete and that the relationships between the variables in the model are correctly specified. If some variables or interactions are omitted, explanations should be given why this is acceptable and does not invalidate the results.

Parameters	

Parameters obtained from experts	The process of parameter elicitation should be described (number of experts, their areas or expertise, questions asked, how the responses were converted to a parameter). Plausibility of the parameter value(s) should be assessed by independent experts. Comparisons should be made with other sources (if available) and the differences explained.

Parameters obtained from the literature	Quality of the source should be ascertained. If available, a published meta-analysis should be used, but a single high-quality study may be an alternative. If information from several sources is combined, the methodology should be explained. Comparisons should be made with alternative sources and discrepancies explained. If alternative sources are not available, plausibility of the parameter values should be assessed by independent experts.

Parameters obtained from data analysis	Validity evidence regarding the data and methods of analysis should be equivalent to that required for a publication in a scientific peer-review journal. The results should be compared with estimates from other sources and (if not available) expert opinion. Evidence to support generalizability of the parameters to the population modeled should be provided.

Parameters obtained through calibration	Calibration methodology should be reported in detail (target data, search algorithm, goodness-of-fit metrics, acceptance criteria, and stopping rule). Plausibility of the parameters derived through calibration should be evaluated by independent experts and their values compared with external data (if available).

Computer implementation	

Selection of model type	A justification for the selected model type should be provided (stochastic vs. deterministic, micro vs. macro-level simulation; discrete vs. continuous time models, interacting agents vs. non-interactive models, etc). Whether or not the type of model is appropriate should be determined by independent experts.

Simulation software	Information should be provided on the simulation software and programming language. The choice of software/language should be justified.

Computer program	Independent experts should evaluate the key programming decisions and approaches used. The results of debugging tests should be documented and the equations underlying the model should be made open to scrutiny by external experts.

Evidence from examining model performance	

Output plausibility	Plausibility (face validity) should be evaluated by subject-matter experts for a wide range of input conditions and output variables, over varying time horizons.

Internal consistency	Internal consistency should be assessed by considering functional and logical relationships between different output variables. Internal consistency should be tested under a wide range of conditions, including extreme values of the input parameters.

Parameter sensitivity analysis	Model validation should include uncertainty and sensitivity analyses of key parameters. Screening methods should be used to select the most influential parameters for more extensive analysis. If feasible, probabilistic uncertainty/sensitivity analysis is recommended. If parameters are estimated through calibration, the model should be recalibrated as part of uncertainty/sensitivity analysis. In probabilistic models, the Monte Carlo error should be estimated.

Between-model comparisons	Comparing the results of different models provides important evidence of validity. Between-model comparisons should take into account the extent to which models are developed independently. If feasible, the impact of different elements of model structure, assumptions, and computer implementation on the results should be evaluated in a systematic fashion.

Comparisons with external data	Ideally, prospective data should be used for external validation. If prospective validation is not feasible, ex-post forecasting and backcasting based on historical data should be used to support predictive validity. Data used for validation should be different from data used in model development and calibration. Cross-validation and bootstrap methods can be considered as an alternative. Criteria for model acceptability should be specified in advance.

Evidence from examining the consequences of model-based decisions	

Quality of decisions	Quality of decisions based on the model should be evaluated and compared with those based on alternative approaches to decision making, using both subjective and objective criteria.

Model usefulness	Uptake of a given model by policy makers should be monitored to assess model usefulness.

The focus of this review has been on validation methods applicable to epidemiological simulation models of non-communicable diseases. Although most of the guidelines should be helpful in evaluating other types of models, including biological models and models of infectious diseases in which the simulated units interact, a limitation of the current review is that additional issues, specific to the latter models, are not discussed.

The importance of input data for model credibility has been discussed extensively in the literature. However, issues pertaining to different data sources for the parameters, calibration, and computer implementation of the model have not been fully addressed in published validation guidelines. Literature on the relative merits of different calibration methods and different types of simulation software is only beginning to emerge. Making the mathematical equations underlying the model available for assessment by independent experts would improve model transparency and hence credibility.

When examining model performance, it is important to assess plausibility of the output and to perform internal consistency and parameter sensitivity analyses. However, the complexity of modern population-based microsimulation models makes sensitivity analysis a challenging and time-consuming task. Usually, sensitivity analyses will be limited to a relatively small subset of parameters selected based on expert opinion and sensitivity screening tools. Important evidence of validity can be obtained by comparing the results between different models, although such evidence is rarely available at this time. As the number of models increases, between-model comparisons may become more common.

With respect to comparing model output with external data, published guidelines are not entirely consistent. It is important in this context to distinguish between decision-analytical models, whose sole purpose is to help with decision making, and explanatory or predictive models that may be used to explain or project trends in health outcomes. For example, POHEM-based models have been used mainly to compare alternative policy scenarios for decision-analytical purposes. However, as population-based models, they are expected to produce outcomes that are useful for explanatory and predictive purposes.

Most authors agree that a failure to predict past or future trends does not automatically disqualify a model from being a useful aid to decision making. The reason is that policy decisions are based on comparisons between different scenarios, in which systematic errors in absolute predictions tend to cancel out. While credibility of both types of models can be enhanced by comparisons with external data that have not been used in model development, such comparisons are especially important for explanatory and predictive models. More research is needed on the use of cross-validation and bootstrap techniques for disease simulation models. Finally, there is a need for further development of validation methods that compare the results of decisions based on different models.

## Competing interests

The authors declare that they have no competing interests.

## Authors' contributions

The manuscript is a result of numerous discussions between the authors and collaboration on research projects in which the issues presented in the manuscript were addressed. JAK conceived the study, reviewed the literature, and drafted the manuscript; PF helped develop the conceptual framework and participated in reviewing the literature and drafting the manuscript; DGM contributed to the conceptual framework, helped review the literature, and participated in drafting the manuscript; DLB participated in conceptualizing and drafting the manuscript; WMF participated in conceptualizing and drafting the manuscript; JO participated in conceptualizing and drafting the manuscript; MA provided methodological support and drafted parts of the manuscript; SH participated in conceptualizing and drafting the manuscript; BS participated in conceptualizing and drafting the manuscript; AO provided methodological support and participated in drafting the manuscript; ECS provided methodological support and participated in drafting the manuscript; MMR provided methodological and data analytical support; MCW participated in conceptualizing the manuscript and provided general oversight and support. All authors have read and approved the final manuscript.

## Appendix

The Appendix can be found within Additional File 1.

## Pre-publication history

The pre-publication history for this paper can be accessed here:

http://www.biomedcentral.com/1471-2458/10/710/prepub

## Supplementary Material

Additional file 1 Optional scoring for the assessment of the degree of model validation. Click here for file
