# Supplementary material for: Validation of population-based disease simulation models: a review of concepts and methods
Source: BMC Public Health. 2010 Nov 18;10:710. doi: 10.1186/1471-2458-10-710 (PMC3001435; doi:10.1186/1471-2458-10-710)
Supplement: Additional file 1 — Optional scoring for the assessment of the degree of model validation. [file 1471-2458-10-710-S1.DOC]

**Appendix**

*Optional scoring for the assessment of the degree of model validation*

The proposed scoring method is based on the recommendations summarized in Table 2. For each section, the extent of validation can be assessed on a scale from 0 to 2 where 0=none, 1=partial, and 2=complete. Sections that are not applicable, given the purpose and structure of the model, should be omitted. For example, section 4 would not apply to models that do not use parameters obtained from experts and section 16 would not apply to models that are not used as aids to decision-making. Scores can be added up and divided by the maximum total score to obtain a percent score. Maximum total score is the sum of maximum possible scores for those sections that are applicable to a given model or application.

The following checklist may be used to calculate the scores:

| Evidence from examining model development process | |
| --- | --- |
| Conceptual model | |
| 1. Underlying theories | 0=none; 1=partial; 2=complete |
| 2. Definitions of variables | 0=none; 1=partial; 2=complete |
| 3. Model content and structure | 0=none; 1=partial; 2=complete |
| Parameters | |
| 4. Parameters obtained from experts | 0=none; 1=partial; 2=complete |
| 5. Parameters obtained from the literature | 0=none; 1=partial; 2=complete |
| 6. Parameters obtained from data analysis | 0=none; 1=partial; 2=complete |
| 7. Parameters obtained through calibration | 0=none; 1=partial; 2=complete |
| Computer implementation | |
| 8. Selection of model type | 0=none; 1=partial; 2=complete |
| 9. Simulation software | 0=none; 1=partial; 2=complete |
| 10. Computer program | 0=none; 1=partial; 2=complete |
| Evidence from examining model performance | |
| 11. Output plausibility | 0=none; 1=partial; 2=complete |
| 12. Internal consistency | 0=none; 1=partial; 2=complete |
| 13. Parameter sensitivity analysis | 0=none; 1=partial; 2=complete |
| 14. Between-model comparisons | 0=none; 1=partial; 2=complete |
| 15. Comparisons with external data | 0=none; 1=partial; 2=complete |
| Evidence from examining the consequences of model-based decisions | |
| 16. Quality of decisions | 0=none; 1=partial; 2=complete |
| 17. Model usefulness | 0=none; 1=partial; 2=complete |
